# *Salmonella enterica* in Invasive Lizard from Fernando de Noronha Archipelago: Serotyping, Antimicrobial Resistance and Molecular Epidemiology

**DOI:** 10.3390/microorganisms8122017

**Published:** 2020-12-17

**Authors:** Carlos R. Abrahão, Luisa Z. Moreno, Jean C. R. Silva, Nilson R. Benites, Carlos E. C. Matajira, Fernando Ferreira, Andrea M. Moreno, Ricardo A. Dias

**Affiliations:** 1 National Center for Conservation of Reptiles and Amphibians, Instituto Chico Mendes de Conservação da Biodiversidade, Brazilian Ministry of Environment, Rua 229, 95, Setor Leste Universitário, 74605 090 Goiânia/GO, Brazil; 2 Laboratory of Epidemiology and Biostatistics, Department of Preventive Veterinary Medicine and Animal Health, School of Veterinary Medicine and Animal Science, University of São Paulo—Av. Prof. Dr. Orlando Marques de Paiva, 87, Cidade Universitária, 05508 270 São Paulo/SP, Brazil; fferreir@usp.br (F.F.); ricardodias@usp.br (R.A.D.); 3 Laboratory of Swine Health, Department of Preventive Veterinary Medicine and Animal Health, School of Veterinary Medicine and Animal Science, University of São Paulo—Av. Prof. Dr. Orlando Marques de Paiva, 87, Cidade Universitária, 05508 270 São Paulo/SP, Brazil; luzanolli@gmail.com (L.Z.M.); k.rlos89.cabrera@gmail.com (C.E.C.M.); morenoam@usp.br (A.M.M.); 4 Department of Veterinary Medicine, Federal Rural University of Pernambuco—Rua Dom Manuel de Medeiros, Dois Irmãos, 52171 900 Recife/PE, Brazil; jcrsilva16@gmail.com; 5 Laboratory of Infectious Diseases, Department of Preventive Veterinary Medicine and Animal Health, School of Veterinary Medicine and Animal Science, University of São Paulo—Av. Prof. Dr. Orlando Marques de Paiva, 87, Cidade Universitária, 05508 270 São Paulo/SP, Brazil; benites@usp.br

**Keywords:** island, genotyping, one health, reservoir, *Salmonella enterica*, *Salvator merianae*

## Abstract

*Salmonella* infection can pose serious health issues, especially to children, elders or immunosuppressed humans. Wild populations of reptiles can reach *Salmonella* prevalence of up to 100% and the direct or indirect transmission from reptiles to humans have been extensively reported. Fernando de Noronha (FN) is an inhabited oceanic archipelago in the northeast coast of Brazil, with an economy based on tourism. The tegu (*Salvator merianae*) is the largest lizard native to South America and was introduced to the archipelago in the early 20th century. This study determines the prevalence, serotypes, antimicrobial resistance, and molecular epidemiology of *Salmonella enterica* in the tegu population from FN archipelago. Results show that *S. enterica* is widely distributed in the FN tegu population, with 43.8% prevalence. The bacteria were isolated from 70.5% of the sampled sites and a total of 15 serotypes were detected in 98 *S. enterica* isolates. Strains were further classified into 31 genotypes. Recaptured animals presented distinct genotypes in each season, demonstrating a seasonal strain turnover. Most *S. enterica* isolates from FN tegus presented low antimicrobial resistance. This is possibly due to geographical isolation of the island population, hampering contact with strains from livestock from the continent, where antimicrobial resistance is common.

## 1. Introduction

*Salmonella* infection is a major source of gastrointestinal disease in humans, especially children, elders or immunosuppressed individuals [1,2]. Reptiles are common asymptomatic reservoirs of *Salmonella*, while the bacteria retain the pathogenicity for warm blooded animals [1]. Food of animal origin are considered to be the main sources of *Salmonella* infection [3], but human infection can also occur when keeping reptiles as pets or through contaminated soil or water [3,4,5]. Wild populations of lizards can reach *Salmonella* prevalence of up to 100% [6,7], thus bearing a high potential of contamination to other species. Aside from the great importance to public health, there’s also the possibility of impact upon the native fauna such as *Salmonella*-related mortality in birds [7].

**Figure 1:**
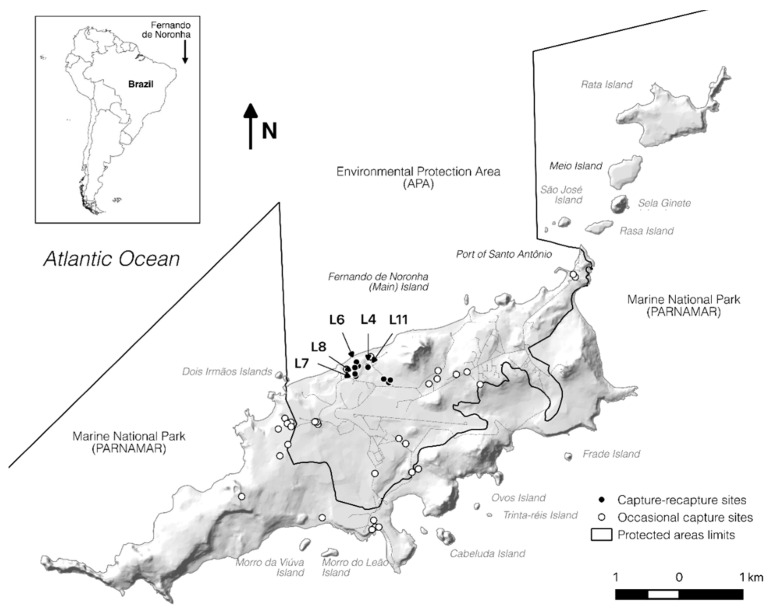
Fernando de Noronha archipelago. Hollow dots = occasional captures sites, filled dots = capture-recapture sites. L4, L6, L7, L8, L11 correspond to the location sites with higher *Salmonella* sp. prevalence.

The Brazilian archipelago of Fernando de Noronha is located 340 km offshore from the northeast South American coast and consists of 21 islands and islets. Total land area of the archipelago is 18 km^2^ where the main island, also named Fernando de Noronha (FN) is about 16.7 km^2^ wide (Figure 1). The archipelago is a UNESCO world heritage site and has recently been named as a Ramsar site, being visited by over 90 thousand tourists every year [8]. Urbanized areas are restricted to the main island and inside the environmental protected area (APA), but the total number of inhabitants and tourists can reach up to eight thousand people in the peak season [9]. The remainder of the main island, including the other islands and islets from the archipelago, is uninhabited and constitutes the National Park (PARNAMAR), where only indirect use is permitted.

The black and white tegu lizard (*Salvator merianae*), hereby referred to as tegu, was deliberately introduced to the main island of FN at the beginning of the 20th century [10], where it is currently considered an invasive exotic species. This diurnal and omnivorous species is the largest endemic lizard of the mainland South America where is commonly seen living and feeding close to inhabited areas [11,12,13,14,15]. In most areas where the tegu occurs, they are hunted for their skin and meat [11,16], which has warranted the inclusion of the species on the CITES II appendix [17]. The tegu population in FN was recently estimated as being between seven thousand and twelve thousand individuals [18].

The aims of this study were to determine the prevalence, serotypes, antimicrobial resistance, and molecular epidemiology of *Salmonella enterica* in the tegu population of Fernando de Noronha archipelago, Brazil.

## 2. Materials and Methods

### 2.1. Sampling

The sampling locations were defined to enable the assessment of the tegu population throughout the island, considering low, medium, and high human usage. Sampling periods were determined based on literature data about the mainland tegu, known to hibernate during the autumn–winter seasons [19,20].

Samples were obtained between 2015 and 2016, during five sampling periods of 2–3 weeks of consecutive daily sampling in the main island of the archipelago. Sample periods were at the end (Jan–Feb) and beginning (Oct–Nov) of the dry season. Animals were captured using Tomahawk^®^ and PVC funnel traps, as previously described elsewhere [18], covering areas with low, medium, and high human use. Snout vent length (SVL) was measured to the nearest 0.5 cm, with the use of a tape measure. The weight was taken using a Pesola^®^ scale with 10 g precision. Captured animals were individually marked with a transponder implanted subcutaneously, prior to release. Recaptured individuals in the same sampling season were promptly released and no data or samples were collected. Recapture efforts were done in the same locations every season, so only part of the sampled population was prone to be recaptured.

Captured animals had the cloacal region cleaned with cotton soaked in chlorhexidine alcoholic solution at 0.5% (Riohex^®^) prior to sample collection. Swabs were introduced in the cloaca avoiding contact with external region. The samples were kept in Stuart’s medium and refrigerated at 4 °C until sent to the laboratory at the end of each campaign (up to three weeks after being obtained).

This study was taken under SISBIO permit no. 41,682 and USP ethics committee no. 2724150515 (approved 17 November 2015).

### 2.2. Salmonella Isolation

The cloacal swabs were directly plated in Xylose Lysine Tergitol 4 (XLT4) agar (Difco—BBL, Sparks, MD, USA), incubated in aerobiosis at 37 °C for 24–48 h. In parallel, samples were also inoculated in tetrathionate broth and incubated at 37 °C for 24 h, and then plated in XLT4 (Difco) and incubated at 37 °C for 24–48 h. One colony of *Salmonella* spp. from each sample, previously identified by biochemical tests, was retrieved for further phenotypic and genotypic characterization. The *Salmonella* isolates were maintained at –80 °C until further serological and molecular typing.

### 2.3. Serotyping

The antigenic characterization of *Salmonella* spp. was obtained using the fast agglutination technique based on the antigenic formulas for *Salmonella* [21] at the Enteric Pathogens Laboratory from Oswaldo Cruz Institute Foundation (FIOCRUZ-RJ).

### 2.4. Molecular Characterization

Purified DNA was recovered according to Boom et al. [22] protocol and stored at −20 °C. *Salmonella* suggestive colonies were confirmed through *invA* gene amplification as previously described [23].

The single-enzyme amplified fragments length polymorphism (SE-AFLP) was performed according to McLauchlin et al. [24] protocol. DNA fragments were detected through electrophoresis at 24 V for 26 h in 2% agarose gel stained with BlueGreen^®^ (LGC Biotecnologia, São Paulo, Brazil) and images were captured under UV illumination by Gel Doc XR System (Bio-Rad Laboratories, Hercules, CA, USA). Molecular weight determinations were done using the 100 bp DNA Ladder (New England BioLabs Inc., Ipswich, MA, USA).

The colistin resistant strains were further assessed for *mcr* genes (*mcr*-1 to *mcr*-5) by PCR using Lescat et al. [25] protocol. The PCR reactions contained 200 μM of each primer, 10× PCR buffer, 1.5 μM MgCl_2_, 200 μM dNTPs, and 1.25 U of Taq polymerase. Amplified fragments were detected by agarose gel (1.5%) electrophoresis using BlueGreen ™ (LGC Biotecnologia, São Paulo, Brazil) and 100 bp DNA ladder (New England BioLabs Inc., Ipswich, MA, USA).

### 2.5. Antimicrobial Susceptibility Profiling

The minimal inhibitory concentration (MIC) was determined by broth microdilution technique, as recommended by the Clinical and Laboratory Standards Institute supplement VET08 [26], using a panel of 18 selected antimicrobials: Ceftiofur, Amoxicillin/Clavulanate, Ampicillin, Meropenem, Fosfomycin, Oxytetracycline, Chloramphenicol, Florfenicol, Nalidixic Acid, Ciprofloxacin, Marbofloxacin, Gentamicin, Neomycin, Azithromycin, Colistin, Sulfamethoxazole, and Trimethoprim/Sulfamethoxazole (MilliporeSigma, St. Louis, MO, USA). *Staphylococcus aureus* ATCC 29,213 was used as internal quality control.

### 2.6. Statistical Analysis

The distribution of strains frequencies according to origin, serotype, resistance, and SE-AFLP profile was performed with SPSS 16.0 (SPSS Inc., Chicago, IL, USA). The multidrug resistance, MIC50 and MIC90 values for the respective antimicrobials were determined according to Schwarz et al. [27].

SE-AFLP results were analyzed with Bionumerics 7.6 software (Applied Maths NV, Saint-Martens-Latem, Belgium). Fingerprint patterns were analyzed by a comprehensive pairwise comparison of restriction fragment sizes, using the Dice coefficient. The mean values obtained from Dice coefficients were employed in UPGMA (unweighted pair group method with arithmetic mean) to generate a dendrogram. A cut-off value of 90% of genetic similarity was applied to analyze the resulting clusters [28], and the discriminatory index was calculated as previously described by Hunter and Gaston [29].

## 3. Results

A total of 153 animals were captured, between 2015 and 2016, on 44 different sites in the main island of FN (Figure 1). Although tegu is present in the Rata Island—as verified through indirect signs—our efforts returned no captures there. The capture locations were classified according to the human use to facilitate understanding the exposure risk.

From the 153 captured animals, 62 were female, 88 were male and in three individuals the gender could not be determined. One hundred and fifteen individuals from the 14 fix sites (filled dots—Figure 1) were captured, marked, and released. Thirty-eight animals from 30 occasional sites (hollow dots—Figure 1) were collected. From the released animals, 26 were recaptured in two different periods, seven were recaptured in three different periods, and only one was recaptured in four out of five collects; a total of 196 cloacal samples taken. From 153 studied animals, 67 (43.8%) were positive for *Salmonella* isolation in at least one sample.

From 196 samples taken, 98 (50.0%) were positive to *Salmonella* sp. Although 70.5% (31/44) of sampled locations were positive for *Salmonella* isolation, half of the isolates originated from only four sites (L6, L4, L11, L8). Most isolates were obtained from collects 1 and 4 (C1 and C4) (30.6% and 23.5%, respectively), followed by C5 with 20.4%, C3 with 17.3%, and the remaining 8.2% originated from C2 collect.

All isolates were identified as *Salmonella enterica* subsp. *enterica* by PCR. Only 12.2% of isolates were not able to be classified by serotyping (Table 1). Rubislaw and Javiana were the most frequent serotypes (13.3% and 12.2%, respectively), followed by Mbandaka, Panama, and Muenchen with 9.2% each, and Minnesota with 7.1%, comprising 58.2% of studied isolates. It is highlighted that only serotype Rubislaw was detected in all five collects (Table 1). 

**Figure 2:**
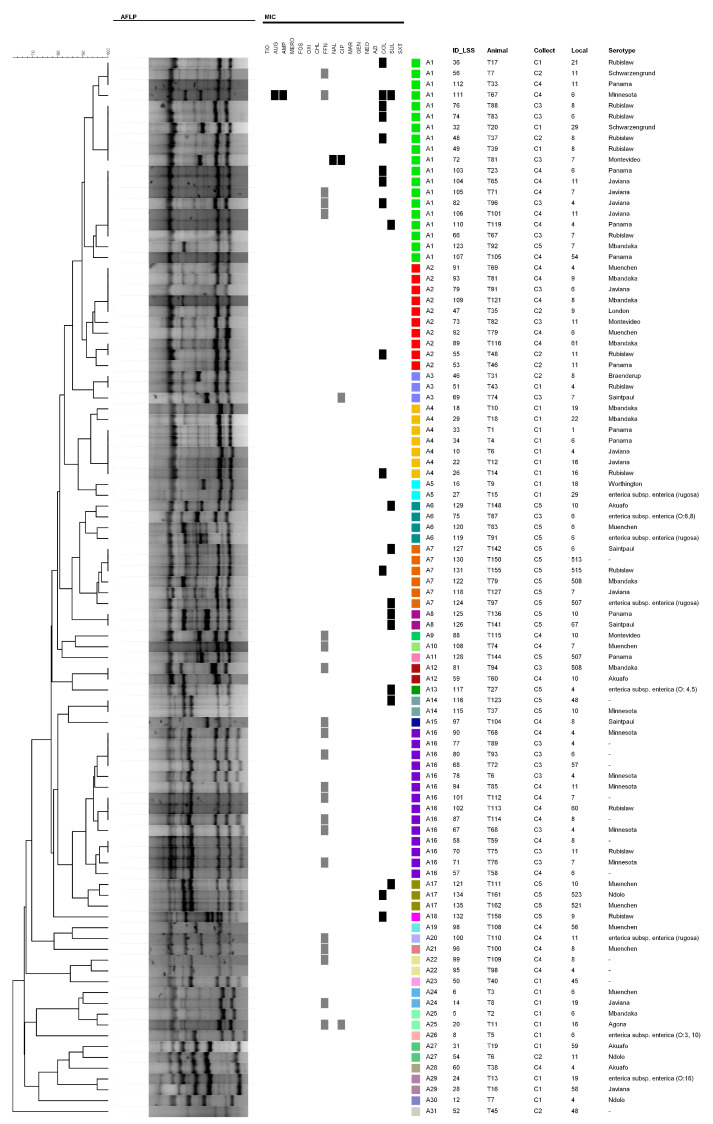
Dendrogram showing the relationship among single-enzyme amplified fragments length polymorphism (SE-AFLP) genotypes and antimicrobial resistance profiles of *S. enterica* strains from tegus of Fernando de Noronha.

Genotyping by SE-AFLP resulted in 31 profiles (A1–A31) (Figure 2). Cluster analysis of fingerprint patterns enabled differentiation of one main group composed by 80 isolates, from 18 genotypes (A1–A18), with over 75% genetic similarity. The remaining 18 isolates presented higher genetic heterogeneity and were distributed among 13 SE-AFLP profiles, and most of them were isolated in the first collect (C1). Three genotypes (A1, A2, and A16) comprised 42.9% of the 98 studied isolates.

There was no clear tendency of clustering according to isolates origin or serotype. Nevertheless, there is a slight tendency to cluster according to the collects, a portion from A1 and A2 genotypes that present higher variety of origin. The discriminatory index obtained for SE-AFLP technique was 0.92. Only two sets of isolates from recaptured animals presented persistence of SE-AFLP profiles (T67 and T68), while most isolates from recaptured animals presented distinct genotypes at each collect (Table 2).

All isolates were susceptible to ceftiofur, meropenem, fosfomycin, oxytetracycline, chloramphenicol, marbofloxacin, gentamycin, neomycin, azithromycin, and trimethoprim/sulfamethoxazole (Table 3).

**Figure 3:**
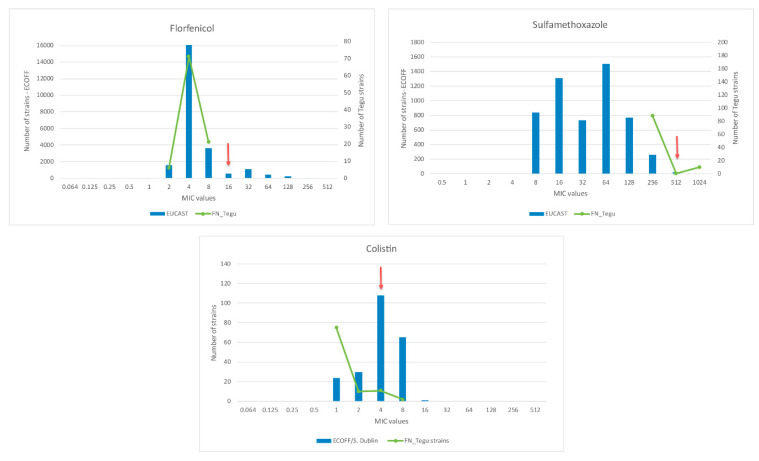
Distribution of *S. enterica* MICs for florfenicol, sulfamethoxazole, and colistin in Fernando de Noronha. Bars correspond to European Committee on Antimicrobial Susceptibility Testing (EUCAST) data of the respective antimicrobials for *Salmonella enterica*; lines correspond to Fernando de Noronha tegu’s *S. enterica* MICs; arrows correspond to antimicrobials resistance breakpoints.

Interestingly, 13.3% of isolates were resistant to colistin, 10.2% to sulfamethoxazole, and 21.4% presented intermediate susceptibility to florfenicol. Only one isolate also presented resistance to ampicillin and amoxicillin/clavulanate, while other was resistant solely to nalidixic acid and ciprofloxacin; a total of 55 isolates (56.1%) were susceptible to the 18 tested antimicrobials. The observed MIC values for colistin, sulfamethoxazole, and florfenicol were further compared to the EUCAST (European Committee on Antimicrobial Susceptibility Testing) MIC distributions and epidemiological cut-off values (ECOFFs) (https://mic.eucast.org/Eucast2/) for *Salmonella enterica* (Figure 3).

## 4. Discussion

The high *Salmonella* prevalence (43.8%) observed among wild tegu corroborates the literature for *Salmonella* occurrence in captive reptiles worldwide [30,31,32,33,34] and the few Brazilian studies that report up to 100% positivity for captive tegus in two Brazilian States [6,35].

At the time of this study, only one isolate of each sample was selected for further analyses; this could represent a drawback considering the possibility of *Salmonella* heterogeneity with the studied animals. Nevertheless, it is interesting that all studied isolates were identified as *Salmonella enterica* subsp. *enterica*. Although reptiles have been described as hosts of a wide variety of *Salmonella enterica* subspecies and exotic serotypes [5,33,36], the predominance of subspecies enterica demands attention for the zoonotic potential as its related serotypes have been commonly isolated from human, alimentary, and environmental sources.

SE-AFLP analysis resulted in high genetic variability among *S. enterica* from wild tegu, with genetic profiles comprising isolates from different serotypes. Even though genetic diversity of reptile *Salmonella* has been poorly assessed, pulsed field gel electrophoresis (PFGE) typing has already demonstrated inter- and intra-serotype heterogeneity. Franco et al. [37] reported genetic similarity between serotypes (Newport and Bardo) and variation of *S.* Pomona within nine pulsotypes among *Salmonella* isolated from land iguanas in an Ecuador island. Similarly, Bertelloni et al. [38] also reported different pulsotypes among *Salmonella* strains from the same serotype isolated from healthy pet reptiles in pet shops in Italy.

In this study, genetic heterogeneity was observed not only among *Salmonella* serotypes but also between the studied areas of FN island. Considering the landscape and that tegus in FN have a home range of up to 15 hectares [18], the detection of the same genotype in distant capture areas at the same sampling season suggests other species could be involved in *Salmonella* dissemination throughout the island. Humans, birds [39,40] or domestic animals could be involved on this transmission chain, but further studies are required to stablish the complete *Salmonella* dynamics in FN.

The large number of different serotypes detected with only a few representatives of each serotype corroborates previous reports of high diversity of *Salmonella* serotypes among reptiles [5,32,33,41]. Most of the serotypes detected in wild tegus had already been reported in reptiles worldwide, of which Agona, Braenderup, Panama, Rubislaw, Saintpaul, and Worthington serotypes had been previously detected in Brazilian captive tegus [6,35]. As previously stated, most of these serotypes have also been identified in humans, birds (including poultry), pigs, and cattle [40].

In contrast to the literature, the *S. enterica* isolates from FN wild tegus presented low prevalence of antimicrobial resistance. Chen et al. [42] and Hossain et al. [43] have reported high drug resistance rates in *S. enterica* isolated from reptiles and eggs in Asia, including over 30% of cephalosporin resistance. In opposition, our study finds 56.1% of the isolates were susceptible to all tested antimicrobials. This found suggests a geographically isolated population that is not in contact with strains common to domestic animals in the continent, where antimicrobial resistance is common in *S. enterica*. Isolation has also been genetically demonstrated for rat populations in Fernando de Noronha and Rata islands in FN archipelago [44].

Interestingly, resistance to colistin and sulfamethoxazole, and intermediate susceptibility to florfenicol were detected among studied isolates. However, Figure 3 demonstrates that when our MIC values are compared to the EUCAST data the MICs distributions are very similar to the values described in the wild type *Salmonella* strains (not exposed to antimicrobials). In addition, the identified colistin resistant strains were further assessed for *mcr* genes by PCR, and all were negative for *mcr*-1 to *mcr*-5 genes presence.

Considering that the archipelago receives tourists of all age groups year-round, the identification of wild tegus as spreaders of *Salmonella enterica* subsp. *enterica* serotypes is of great concern to public health. Moreover, it should be noted that tegu lizards are not used as a food source in FN. From a conservation perspective, adding commercial value to tegu in FN (e.g., using it as a protein source) will likely create dependence on this resource by the local community, creating further obstacles to any control or eradication program in the future and perpetuating the establishment of tegu in FN archipelago.

## Figures and Tables

**Table 1:** Distribution of detected serotypes among sampling periods—N (%).

Serotype	Collect					Total
	C1 October/2014	C2 February/2015	C3 October/2015	C4 February/2016	C5 October/2016	
**Rubislaw**	4 (17.4)	2 (25.0)	4 (23.5)	1 (3.3)	2 (10.0)	13 (13.3)
**NT**	0	1 (12.5)	3 (17.6)	6 (20.0)	2 (10.0)	12 (12.2)
**Javiana**	4 (17.4)	0	2 (11.8)	3 (10.0)	1 (5.0)	10 (10.2)
**Mbandaka**	3 (13.0)	0	1 (5.9)	3 (10.0)	2 (10.0)	9 (9.2)
**Panama**	2 (8.7)	1 (12.5)	0	4 (13.3)	2 (10.0)	9 (9.2)
**Muenchen**	1 (4.3)	0	0	5 (16.7)	3 (15.0)	9 (9.2)
**Minnesota**	0	0	3 (17.6)	3 (10.0)	1 (5.0)	7 (7.1)
***S. enterica* subsp. enterica (rugose)**	2 (8.7)	0	0	1 (3.3)	2 (10.0)	5 (5.1)
**Akuafo**	1 (4.3)	0	0	2 (6.7)	1 (5.0)	4 (4.1)
**Saintpaul**	0	0	1 (5.9)	1 (3.3)	2 (10.0)	4 (4.1)
**Montevideo**	0	0	2 (11.8)	1 (3.3)	0	3 (3.1)
**Ndolo**	1 (4.3)	1 (12.5)	0	0	1 (5.0)	3 (3.1)
**Schwarzengrund**	1 (4.3)	1 (12.5)	0	0	0	2 (2.0)
**Agona**	1 (4.3)	0	0	0	0	1 (1.0)
**Braenderup**	0	1 (12.5)	0	0	0	1 (1.0)
**London**	0	1 (12.5)	0	0	0	1 (1.0)
**Worthington**	1 (4.3)	0	0	0	0	1 (1.0)
***S. enterica* subsp. enterica (O: 4,5)**	0	0	0	0	1 (5.0)	1 (1.0)
***S. enterica* subsp. enterica (O:16)**	1 (4.3)	0	0	0	0	1 (1.0)
***S. enterica* subsp. enterica (O:3,10)**	1 (4.3)	0	0	0	0	1 (1.0)
***S. enterica* subsp. enterica (O:6,8)**	0	0	1 (5.9)	0	0	1 (1.0)
**Total**	23 (100)	8 (100)	17 (100)	30 (100)	20 (100)	98 (100)

**Table 2:** Assessment of recaptured animals among sampling periods according to isolation sites and AFLP genotypes (cells are colored according to isolation sites).

Animal	Data	Collect				
		C1 October/2014	C2 February/2015	C3 October/2015	C4 February/2016	C5 October/2016
**T4**	Site	**L6**	**L11**			
	AFLP profile	**A4**	Negative			
**T6**	Site	**L4**	**L11**	**L4**		
	AFLP profile	**A4**	**A27**	**A16**		
**T7**	Site	**L4**	**L11**			
	AFLP profile	**A30**	**A1**			
**T23**	Site		**L5**		**L6**	
	AFLP profile		Negative		**A1**	
**T27**	Site		**L4**		**L4**	
	AFLP profile		Negative		**A13**	
**T37**	Site		**L8**			**L10**
	AFLP profile		**A1**			**A14**
**T65**	Site			**L11**	**L11**	
	AFLP profile			Negative	**A1**	
**T67**	Site			**L7**	**L6**	
	AFLP profile			**A1**	**A1**	
**T68**	Site			**L4**	**L4**	
	AFLP profile			**A16**	**A16**	
**T69**	Site			**L4**	**L4**	
	AFLP profile			Negative	**A2**	
**T71**	Site			**L8**	**L7**	
	AFLP profile			Negative	**A1**	
**T74**	Site			**L7**	**L7**	
	AFLP profile			**A3**	**A10**	
**T76**	Site			**L7**		**L508**
	AFLP profile			**A16**		Negative
**T79**	Site			**L9**	**L6**	**L508**
	AFLP profile			Negative	**A2**	**A7**
**T81**	Site			**L7**	**L9**	**L5**
	AFLP profile			**A1**	**A2**	Negative
**T83**	Site			**L6**		**L6**
	AFLP profile			**A1**		**A6**
**T85**	Site			**L11**	**L11**	
	AFLP profile			Negative	**A16**	
**T87**	Site			**L6**	**L6**	
	AFLP profile			**A6**	Negative	
**T91**	Site			**L6**		**L6**
	AFLP profile			**A2**		**A6**
**T92**	Site			**L7**		**L507**
	AFLP profile			Negative		**A1**
**T93**	Site			**L6**		**L508**
	AFLP profile			**A16**		Negative
**T97**	Site			**L7**		**L507**
	AFLP profile			Negative		**A7**
**T98**	Site				**L4**	**L4**
	AFLP profile				**A22**	Negative
**T111**	Site				**L1**	**L10**
	AFLP profile				Negative	**A17**
**T112**	Site				**L7**	**L10**
	AFLP profile				**A16**	Negative
**T114**	Site				**L8**	**L8**
	AFLP profile				**A16**	Negative
**T121**	Site				**L8**	**L508**
	AFLP profile				**A2**	Negative

**Table 3:** Minimal inhibitory concentration (MIC) range, MIC50, MIC90, and resistance rates of *Salmonella* isolates against tested antimicrobials. S = susceptible; I = intermediate; R = resistant.

Antimicrobial	Range (µg/mL)	S N (%)	I N (%)	R N (%)	MIC50 (µg/mL)	MIC90 (µg/mL)
**Ceftiofur**	0.25–8	98 (100)	0	0	0.5	1
**Amoxicillin/Clavulanate**	1/0.5–32/64	97 (99.0)	0	1 (1.0)	≤1/0.5	≤1/0.5
**Ampicillin**	1–64	97 (99.0)	0	1 (1.0)	≤1	2
**Meropenem**	0.25–8	98 (100)	0	0	≤0.25	≤0.25
**Fosfomycin**	8–512	98 (100)	0	0	≤8	16
**Oxytetracycline**	2–32	98 (100)	0	0	≤2	≤2
**Chloramphenicol**	4–64	98 (100)	0	0	≤8	8
**Florfenicol**	0.5–8	77 (78.6)	21 (21.4)	0	4	8
**Nalidixic Acid**	8–128	97 (99.0)	−	1 (1.0)	≤8	≤8
**Ciprofloxacin**	0.06–8	95 (97.0)	2 (2.0)	1 (1.0)	≤0.06	≤0.06
**Marbofloxacin**	0.06–8	98 (100)	0	0	≤0.06	≤0.06
**Gentamicin**	0.5–32	98 (100)	0	0	≤0.5	2
**Neomycin**	4–16	98 (100)	−	0	≤4	≤4
**Azithromycin**	4–64	98 (100)	−	0	8	16
**Colistin**	1–16	85 (86.7)	−	13 (13.3)	≤1	4
**Sulfamethoxazole**	256–1024	88 (89.8)	−	10 (10.2)	≤256	>1024
**Trimethoprim/Sulfamethoxazole**	2/18–4/76	98 (100)	0	0	≤2/18	≤2/18
